# Evaluating the Methodological Quality of Postexercise Hypotension Aerobic Exercise Interventions

**DOI:** 10.3389/fphys.2022.851950

**Published:** 2022-03-10

**Authors:** Christina Day, Yin Wu, Linda S. Pescatello

**Affiliations:** ^1^Department of Kinesiology, University of Connecticut, Storrs, CT, United States; ^2^Institute for Collaboration on Health, Intervention, and Policy, University of Connecticut, Storrs, CT, United States

**Keywords:** blood pressure, cardiovascular disease, hypertension, physical activity, systematic review

## Abstract

**Background:**

Postexercise hypotension (PEH) is the immediate reduction in blood pressure (BP) of 5–8 mmHg that occurs after a single bout of aerobic exercise among adults with hypertension. Across PEH studies, there are variations in the level of rigor of the study designs and methods that limit the conclusions that can be made about PEH.

**Objective:**

To develop and then apply a methodological study quality evaluation checklist to aerobic exercise PEH studies to provide methodological guidance.

**Methods:**

We developed a PEH checklist (PEH√list) based upon contemporary methodological study quality standards. The PEH√list contains 38 items divided into three categories: sample (*n* = 10 items), study (*n* = 23 items), and intervention characteristics (*n* = 5 items). We then systematically searched six databases to January 2019 to identify and then evaluate studies that: (1) enrolled adults ≥18 years with hypertension and without other chronic diseases or conditions; (2) included a bout of aerobic exercise and a non-exercise control session; and (3) were published in English.

**Results:**

Of 17,149 potential studies, 64 qualified. Participants (*N* = 1,489) were middle-aged (38.6 ± 15.6 year), overweight (26.1 ± 2.5 kg/m^2^) mostly men (64.4%) with elevated BP (systolic BP 129.5 ± 15.2/diastolic BP 81.0 ± 10.1 mmHg). Overall, the qualifying studies satisfactorily reported 53.9 ± 13.3% (24.2–82.8%) of the relevant items on the PEH√list. Of note, only 20.3% of the studies disclosed BP was measured following professional guidelines, 18.8% reported BP was taken by the same assessor pre- and post-intervention, and 35.5% stated participants abstained from caffeine, alcohol, and physical activity prior to testing. Half (51.5%) indicated they statistically controlled for pre-exercise/baseline BP. Meanwhile, 100% of the studies reported the setting in which the BP measurements were taken, time from the end of the exercise to the start of the BP measurements, and if relevant, the length of the ambulatory BP monitoring period.

**Conclusion:**

Overall, the PEH√list items were not well satisfied; especially items with potential confounding effects on PEH. We contend the PEH√list provides guidance to investigators on the important methodological study considerations in PEH aerobic exercise studies that should be attended to in the future.

**Systematic Review Registration:**

[https://www.crd.york.ac.uk/PROSPERO/], identifier [#CRD42020221996].

## Introduction

Cardiovascular disease (CVD) is the leading cause of death in the United States and the world, accounting for approximately one in three deaths annually (Virani et al., 2021). Hypertension is the most common, costly, and preventable CVD risk factor affecting nearly 50% of adults in the United States. The total United States health care expenditures attributed to hypertension in 2016 were $79 billion dollars (Virani et al., 2021) and are projected to be $153.7 billion dollars in 2035 (Nelson et al., 2016) underscoring the public health burden hypertension places on our society.

Professional organizations throughout the world recommend exercise as first-line lifestyle therapy to lower blood pressure (BP; Pescatello et al., 2015a; Whelton et al., 2018). *Postexercise hypotension* (PEH) is the immediate reduction in BP of 5–8 mmHg that occurs after a single bout of aerobic exercise and persists for up to 24 h. PEH is clinically important because: (1) PEH occurs immediately (Fitzgerald, 1981; Pescatello et al., 1991; Kenney and Seals, 1993); (2) PEH reduces BP throughout the day when BP is typically at its highest levels (Pescatello et al., 2004); (3) an individual does not have to be physically fit to experience PEH (Pescatello et al., 2003, 2007, 2017; Ash et al., 2017; Zaleski et al., 2019); and (4) PEH can be used as a behavioral self-regulation strategy to increase exercise adherence (Zaleski et al., 2019). Also, there is evidence that PEH is correlated with the BP response to the exercise training effect (Pescatello et al., 2015a,b; Wegmann et al., 2018).

Within the PEH literature, there is a wide range of variations in the study designs. Some of the variations include: (1) PEH studies may or may not include a control comparison (de Brito et al., 2019); (2) PEH studies may or may not disclose baseline/pre-exercise BP levels (de Brito et al., 2019); (3) PEH studies include different intensities, modalities, and durations of exercise (Pescatello et al., 2015a; US Department of Health and Human Services, 2018); (4) PEH studies involve samples with an admixture of BP status, ranging from normal to stage 2 hypertension (Chobanian et al., 2003; Pescatello et al., 2015a); and (5) BP monitoring occurs in different settings, notably in the laboratory or under ambulatory conditions (Pescatello et al., 2015a; de Brito et al., 2019). Due to the variance in the exercise protocols between studies, it is important for studies to clearly report the intensity, time, and type of the exercise intervention so that the exercise dose that elicits PEH can be more clearly defined (MacDonald, 2002; Pescatello et al., 2015a; de Brito et al., 2019; Fecchio et al., 2020). For example, studies including participants with normal BP will underestimate the magnitude of PEH, as consistent with the law of initial values, those with the highest resting BP will experience the greatest BP reductions resulting from exercise (Wilder, 1965; Eicher et al., 2010; Pescatello et al., 2019; Hanssen et al., 2021). As a result, the 2018 Physical Activity Guidelines Advisory Committee called for additional well-controlled studies to better understand PEH (Pescatello et al., 2015a; US Department of Health and Human Services, 2018).

To the best of our knowledge, there is no existing easy-to-use checklist or scale that researchers can follow when designing, implementing, or reporting PEH studies. Therefore, we have developed a 38-item evaluation instrument named, the *Evaluation Tool for Studies Examining Postexercise Hypotension* or the PEH√list. We then performed a high-quality systematic review to evaluate studies examining the BP response to acute aerobic exercise. Based on our findings, our intent was also to provide methodological guidance to investigators studying PEH.

## Methods

### Development of the PEH√list and Selection of Core Items

We developed an evaluation instrument named, *Evaluation Tool for Studies Examining Postexercise Hypotension* (the PEH√list), consisting of three categories: sample, study, and intervention characteristics. See Supplementary Material A for a complete copy of the PEH√list. We identified the items on the PEH√list based upon our extensive experience conducting PEH studies (Downs and Black, 1998; Higgins et al., 2011; Ash et al., 2013; Johnson et al., 2014; Hacke et al., 2018; de Brito et al., 2019). We also consulted articles regarding general methodological study quality standards for randomized controlled trials that included the Cochrane tool for assessing risk of bias (Higgins et al., 2011) and the Downs and Black checklist for methodological quality (Downs and Black, 1998). We included the specifics of the intervention characteristics such as reporting the frequency, intensity, time, and type of the exercise intervention (Johnson et al., 2014). Last, we also included, methods papers commenting on unique aspects of PEH studies such as de Brito et al. (2019), commented on the different statistical approaches for calculating PEH (Ash et al., 2013; Hacke et al., 2018).

The PEH√list consists of three sections with a total of 38 items: (1) sample characteristics (10 items); (2) study characteristics (23 items); and (3) intervention characteristics (5 items). The total number of relevant items evaluated in the PEH√list for a study was dependent on the method used for measuring BP (i.e., resting BP, ambulatory BP, or both). Accordingly, a total of 38 PEH√list items pertained to a study if both resting BP and ambulatory BP measurements were reported, 29 PEH√list items pertained to a study if only resting BP was reported, and 33 PEH√list items pertained to a study if only ambulatory BP was reported. In addition, we have selected 13 core items that are shaded in gray in Tables 1–3 that we contend are fundamental considerations in designing, implementing, and reporting findings from PEH studies to ensure transparent replication of the methods and trustworthiness of the findings (Guadagnoli and Velicer, 1988; Downs and Black, 1998; Whelton et al., 2018; Flack and Adekola, 2020).

**TABLE 1:** PEH√list part one participant characteristics.

Items	*k*	Reporting rates	
1. Age (year)	61	95.3%	38.6 ± 15.7
2. Ethnicity/race	8	12.5%	–
3. Gender/sex	60	93.8%	Male (*n* = 960, 66.9%) Female (*n* = 476, 33.1%)
4. BP classification scheme used	36	56.3%	
4a. Followed professional guidelines	11	30.6%	American College of Sports Medicine (*k* = 1) Brazilian Guidelines for Hypertension (*k* = 2) World Health Organization (*k* = 2) American Heart Association (*k* = 3) 7th Joint National Committee (*k* = 3)
*Values at Baseline*			
5. BP (mmHg)	41	64.1%	129.5 ± 15.2/81.0 ± 10.1
6. Physical activity level	45	70.3%	Active (46.7%, *k* = 21) Inactive/Sedentary (48.9%, *k* = 22) Mixture Active and Inactive (4.4%, *k* = 2)
7. Cardiorespiratory fitness level (mL/kg.min^–1^)	37	57.8%	35.3 ± 9.9
8. Body mass index (kg/m^2^)	57	89.1%	26.1 ± 2.5
9. Waist circumference (cm)	10	15.6%	86.7 ± 27.9
10. Medication use	53	82.8%	
10a. Reported the type and/or dosage of medication*	39	75.0%	–
10b. Reported the length of the washout or run-in period* (weeks)	11	21.2%	1–6

BP, Blood Pressure. Items shaded in gray are the core items. *k values and percentage is based on the main question.

### Literature Search and Study Screening

**FIGURE 1:**
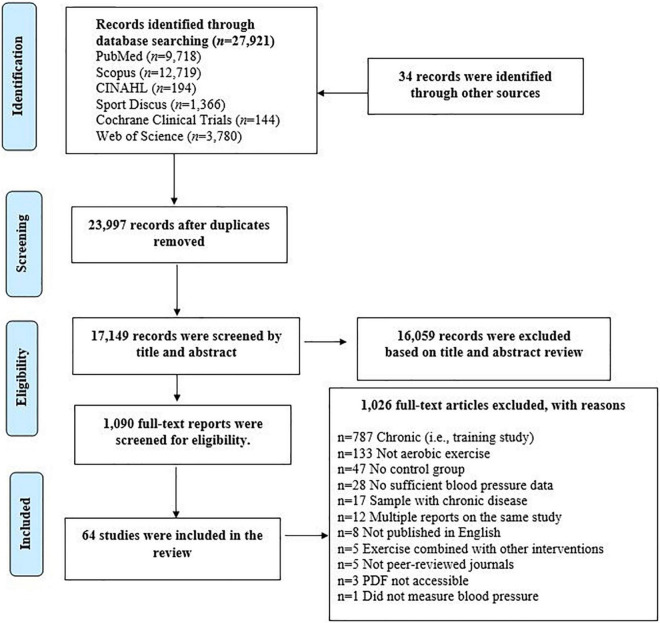
Preferred reporting items for systematic reviews and meta-analyses flow chart detailing the systematic search of potential intervention studies.

This systematic review was conducted according to the Preferred Reporting Items for Systematic Reviews and Meta-Analyses (PRISMA) guidelines (Higgins et al., 2011). The protocol was registered at PROSPERO (#CRD42020221996). Qualifying articles were retrieved from electronic databases (PubMed, Scopus, Sport Discuss, CINAHL, Cochrane, and Web of Science) from inception until January 2019, with key words related to aerobic exercise and BP. Studies qualified if they: (1) were peer-reviewed and published in English; (2) involved healthy adults ≥18 years; (3) included an acute bout of aerobic exercise; and (4) included a non-exercise control session to control for the circadian variation in BP (de Brito et al., 2019). The potentially relevant studies were screened by two trained coders (CD, YW); first by title and abstract, and then by full text. See Figure 1 for the flow diagram and Supplementary Material B for references of included PEH intervention studies. All disagreements were resolved through discussion by two independent reviewers (CD, YW). When an agreement could not be reached, a third party was consulted (LSP).

### Data Extraction and Study Evaluation

Data were extracted using a standardized coding form and coder manual we adapted for PEH studies (Johnson et al., 2014). Coders extracted and entered information regarding the study (e.g., publication year, number of participants, and location), participant (e.g., age, gender, and body mass index), intervention (e.g., exercise intensity, exercise type, and time of day when sessions began) characteristics, and methodological study quality. The risk of bias was assessed in accordance with the Revised Cochrane Risk-of-Bias Tool for Randomized Trials (Higgins et al., 2011). The five domains evaluated for risk of bias were: randomization process, deviations from intended interventions, missing outcome data, measurement of the outcome, and selection of the reported result in each included study. Studies were rated as low, some concern, or high risk of bias. Methodological study quality was assessed using an augmented version of Downs and Black Checklist (Downs and Black, 1998). Methodological study quality was reported as the percentage of items satisfied out of a possible 29 items. The overall methodological quality was classified as: low (<50%), moderate (50–79%), and high (≥80%). We conducted a preliminary correlation analysis which showed the PEH√list study score is correlated with the Downs and Black Checklist score. Therefore, we used the cutoffs in Downs and Black Checklist to define the PEH√list study scores as low (<50%), moderate (50–79%), and high (≥80%). All disagreements were resolved through discussion by two independent reviewers (CD, YW). When an agreement could not be reached a third party was consulted (LSP).

### Statistical Analysis

Descriptive statistics were calculated for the baseline characteristics of the sample in the qualifying studies. For each of the PEH√list items, the reporting rate was calculated as (the number of studies satisfactorily reporting this item/the number of studies to which this item was deemed relevant) × 100%. For each of the studies included, the PEH√list study score was calculated as (the number of items reported/by the number of relevant items) × 100%. We also compared the PEH√list study scores against a validated study quality scale score, the Downs and Black Checklist (Pescatello et al., 2019), by performing a Pearson Correlation test. All analyses were performed using IBM SPSS Statistics for Windows, Version 26.0.

## Results

The initial search resulted in 27,921 potentially qualifying studies. An additional 34 records were identified through manual searches. After triaging, 64 studies qualified. See Figure 1 for the PRISMA flow diagram. The average reporting rate for PEH√list items was 61.8 ± 31.7%.

### PEH√list Part 1: Sample Characteristics

The reporting rate for each of the items in the PEH√list Part 1 is listed in Table 1. The sample (*n* = 1,511) consisted of young to middle-aged (38.6 ± 15.7 years) healthy adults who on average, were overweight (body mass index 26.1 ± 2.5 kg/m^2^) and had no chronic conditions other than hypertension (129.5 ± 15.2/81.0 ± 10.1 mmHg). Over half of the participants were men (66.9%, *n* = 960), and nearly half were physically inactive (48.9%, *k* = 22). In addition, only 12.5% of the studies (*k* = 8) reported the ethnicity/race of the participants. A majority (82.8%, *k* = 53) of the studies controlled for the potential influence of medications that could impact the BP response to exercise. Of these studies, less than half excluded participants if they were taking various medications. Among the studies excluded participants due to medication use, more than half (*k* = 12, 46.2%) excluded participants if they were taking antihypertensive medications; a few (*k* = 4, 15.4%) excluded participants if they were taking antihypertensive medication or oral contraceptive (*k* = 1) and lipid medication (*k* = 3); one study (0.04%) excluded participants who were taking any medication; and the rest (*k* = 9, 34.6%) excluded participants who were taking medications that can alter lipid profile (*k* = 4), metabolism (*k* = 2), heart rate (*k* = 2), and the renin–angiotensin system (*k* = 1). For the remainder of the 53 studies, participants: (1) remained on the same medication throughout the study (11%, *k* = 7); (2) were not taking any medication (12.5%, *k* = 8); or (3) stopped taking medication by going through a washout period of 1–6 weeks before the study started (17.5%, *k* = 11). Of note, only 45.3% (*k* = 29) of the studies identified their participants had hypertension, however, only 30.6% (*k* = 11) of the studies reported following professional guidelines to classify the subjects as having hypertension.

### PEH√ Part 2: Study Characteristics

The reporting rate for each of the items in the PEH√list Part 2 is listed in Table 2. The included studies were mostly randomized controlled trials (95.3%, *k* = 61) published between 1987 and 2018 (2006 ± 8) and conducted in North America (35.9%, *k* = 23), South America (32.8%, *k* = 21), Europe (26.6%, *k* = 17), Asia (3.1%, *k* = 2), and Australia (1.6%, *k* = 1). Studies included 6–109 participants (24 ± 20), and more than half contained multiple exercise arms (64%, *k* = 41), while 15.6% (*k* = 10) contained multiple control arms. Of note, only 25% (*k* = 16) of the studies reported they performed a sample size estimation based on BP as the primary outcome, and only six studies (9.8%) reported the procedure used for randomization.

**TABLE 2:** PEH√list part two study characteristics.

Items	*k*	Reporting %	
11. Performed sample size estimation analysis based on BP as the primary outcome	16	25.0%	–
12. The allocation sequence	61	95.3%	Randomized
13a. Disclosed the procedure used*	5	7.8%	Table with random number (*k* = 1) Used Randomizer.org (*k* = 4)
13. The investigator who performed the BP measurements	5	7.8%	i.e., trained investigator
14. The same investigator performed all BP measurements	12	18.8%	–
15. The model of the BP device	62	96.9%	Ambulatory (*k* = 25, 40.3%), e.g., Accutracker II Automated (*k* = 23, 37.1%), e.g., Microlife Baroreflex Responses (*k* = 1, 1.6%) Finapres (*k* = 5, 8.1%), e.g., Ohmeda Finapres Manuel (*k* = 8, 12.9%), e.g., Mercury Sphygmomanometer
15a. The same BP device used through the study for a participant*	4	6.6%	–
16. Participant abstained from caffeine prior to intervention	40	62.5%	–
16a. Hours participant abstained from caffeine*	29	72.5%	15.6 ± 11.8
17. Participant abstained from alcohol prior to intervention	27	42.2%	–
17a. Hours participant abstained from alcohol*	20	74.1%	21.4 ± 21.2
18. Participant abstained form physical activity prior to intervention	33	51.6%	–
18a. Hours participant abstained form physical activity*	31	93.9%	28.5 ± 12.8
*Was The BP Response to Exercise Controlled for By Pre-Exercise BP*			
19a. Reported Average = (Average BP post-exercise) minus (Average BP post-control) with baseline/pre-exercise BP as a covariate	10	15.6%	–
19b. Reported Change from baseline/pre-exercise BP = (Average BP post- minus pre-exercise) minus (Average BP post- minus pre-control) with or without baseline/pre-exercise BP as a covariate	23	35.9%	–
*Resting Blood Pressure Measurement Protocol*	41		
20. Location/environment	41	100%	Aquatic Center (*k* = 1) Lab and Workplace (*k* = 2) Lab and Outdoors (*k* = 1) Lab (*k* = 37)
21. Followed professional guidelines during BP measurements	7	17.1%	5th Brazilian Guidelines for Hypertension (*k* = 2) 7th Joint National Committee (*k* = 2) American Heart Associations (*k* = 1) Brazilian Society of Cardiology (*k* = 1) International Protocol of the European Society of Hypertension (*k* = 1)
22. Participant’s position	40	97.6%	Seated (*k* = 25) Supine (*k* = 14) Semi recumbent (*k* = 1)
23. Time Lapse from the end of exercise and start of the BP measurements (minutes)	41	100%	12.6 ± 11.0 (2–45)
24. Total time of the BP monitoring (minutes)	38	92.7%	142.1 ± 247.3 (5–1,440)
*Ambulatory Blood Pressure Measurement Protocol*	27		
25. Followed professional guidelines during BP measurements	6	22.2%	American Heart Association (*k* = 2) British Hypertension Society (*k* = 1) European Society of Hypertension (*k* = 1) 5th Brazilian Guidelines for Hypertension (*k* = 1) Brazilian Guidelines for Ambulatory BP Monitoring (*k* = 1)
26. Performed a calibration check	9	33.3%	Calibrated against mercury sphygmomanometer (*k* = 9)
27. Including participant familiarization to wearing the ambulatory BP monitor	6	22.2%	–
28. Participants were given instruction while wearing the BP monitor	22	81.5%	Keep similar routine/No physical activity (*k* = 5) Instructed to keep arm still during measurements (*k* = 17) Instructed to keep an activity log (*k* = 11)
29. Time-lapse from the end of exercise and start of BP measurements (minutes)	24	88.9%	27.1 ± 17.8 (2–100)
30. Location/environment	27	100%	Free-Living Conditions (*k* = 24) Lab and Free-Living Conditions (*k* = 2) Lab (*k* = 1)
31. Disclosed when ABP monitor was attached during the day	7	25.9%	–
32. Total time of the BP monitoring (minutes)	27	100%	1,206 ± 366 (120–1,440)
33. Specified acceptable level of missing data for ambulatory BP analysis	17	63.0%	79.40% ± 14.2 (25–95.6%)

*BP, Blood Pressure. Items shaded in gray are the core items.*

*k values and percentage is based on the main question.

When calculating PEH more than half (51.6%, *k* = 33) of the studies reported controlling for baseline/pre-exercise BP by including baseline/pre-exercise BP as a covariate in the statistical models comparing: (1) average BP post-exercise versus average BP post-control (15.6%, *k* = 10); or (2) the change of BP due to exercise (i.e., post-exercise BP – pre-exercise BP) versus the change of BP due to control (i.e., post-control BP – pre-control BP) (35.9%, *k* = 23).

Regarding the measurement of BP, 64.1% of the studies (*k* = 41) measured resting BP, 42.2% measured ambulatory BP (*k* = 27), or 6.3% measured both (*k* = 4). Among the 41 studies measuring resting BP, most were measured in the seated position (62.5%, *k* = 25) in the laboratory (90.2%, *k* = 37) starting 12.6 ± 11 min after the end of the exercise session and continued for 142.1 ± 247.3 min thereafter. Of the 27 studies assessing ambulatory BP, most occurred under free-living conditions (88.9%, *k* = 24) starting 27.1 ± 17.8 min after the end of the exercise sessions and continued for 20.1 ± 6.1 h. Of note, most studies (96.9%, *k* = 62) reported the model of the BP device used to measure BP. However, studies rarely disclosed they followed protocols recommended by professional guidelines when measuring resting BP (only 17.1% did, *k* = 7) or ambulatory BP (only 22.2% did, *k* = 6). The studies assessing ambulatory BP rarely (only 22.2% did, *k* = 6) disclosed whether a familiarization session was performed prior to the start of experiments, or a calibration check was performed (only 33.3% did, *k* = 9). In addition, only 31.3% (*k* = 20) of the 64 studies assessing resting and/or ambulatory BP asked participants to abstain from physical activity, alcohol, and caffeine prior to experiments.

### PEH√ Part 3: Intervention Characteristics

The reporting rate for each of the items in the PEH√list Part 3 is listed in Table 3. Over half of the studies (53.1%, *k* = 34) reported the time of day of the exercise and control sessions, with most of the sessions occurring in the morning between 7:00 am and 12:00 pm (42.2%, *k* = 27). There were 99 exercise arms in the included studies. The majority of exercises were performed in a laboratory setting (*k* = 44, 88.0%), two studies (4.0%) performed exercises in aquatic setting, and others were in workplace (*k* = 1, 2.0%), thermal bath (*k* = 1, 2.0%), and a combination of laboratory and outdoors (*k* = 1, 2.0%). There were 25 studies (39.1%) that reported the temperature that the participants exercised in with the lowest temperature range being 15–22°C in Casonatto et al. (2011), and the highest temperature range being ≤36.0°C in Matzer et al. (2017). The exercise sessions on average lasted 41.0 ± 22.3 min at moderate (82.8%, *k* = 82) and vigorous (17.2%, *k* = 17) intensity measured by various methods such as peak oxygen uptake, maximal oxygen uptake, and heart rate maximum (see Table 3 for a complete list of intensity methods). Most of the studies were performed on a cycle ergometer (61.6%, *k* = 61) or treadmill (32.3%, *k* = 32). Among the 61 control arms, control sessions on average lasted 48.3 ± 58.6 min with seated rest being the most common (93.2%, *k* = 55) (see more details in Table 3).

**TABLE 3:** PEH√list part three intervention characteristics.

Items	*k*	Reporting %	
34. The time of day the exercise and control sessions began	34	53.1%	Morning 7:00 am–12:00 pm (*k* = 27) Afternoon 12:00 pm–5:00 pm (*k* = 2) Evening 5:00 pm–7:00 pm (*k* = 3) Both morning and evening times (*k* = 2)
34a. The start of exercise and control sessions were conducted within 3–4 h of one another*	37	97.4%	
35. The location of exercise	50	78.1%	Workplace (*k* = 1, 2.0%) Thermal Bath (*k* = 1, 2.0%) Laboratory and Outdoors (*k* = 1, 2.0%) Chamber (*k* = 1, 2.0%) Aquatic (*k* = 2, 4.0%) Laboratory (*k* = 44, 88%)
36. The temperature that participants exercised in	25	39.1%	15–36 Celsius
37. The time, intensity, and type of the exercise intervention*^a^*	58	90.6%	*Time* 41 ± 22.3 min *Intensity* VO_2_peak 56 ± 0.16% (*k* = 37, 37.4%) VO_2_max 61 ± 0.09% (*k* = 20, 20.2%) ml/kg.min 22.815 ± 10.13 (*k* = 10, 10.1%) Heart Rate Max 74.83 ± 0.12% (*k* = 7, 7.1%) Ventilatory Threshold 80% (*k* = 4, 4.0%) Heart Rate Peak 75 ± 0.17% (*k* = 4, 4.0%) Heart Rate Max Age 59 ± 0.02% (*k* = 3, 3.0%) Anaerobic Threshold 100 ± 0.21% (*k* = 2, 2.0%) Rate of Perceived Exertion 15.25 ± 3.18 (*k* = 2, 2.0%) *Type* Cycle Ergometer (*k* = 61, 61.6%) Treadmill (*k* = 32, 32.3%) Aquatic (*k* = 2, 2.0%) Other (*k* = 4, 4.0%)
38. The content of the sham control session*^b^*	49	76.6%	*Time* 48.3 ± 58.6 min *Position* Seated Rest (*k* = 55, 93.2%) Option to Stand or Sit (*k* = 2, 3.3%) Standing (*k* = 1, 1.6%) Supine Rest (*k* = 3, 4.9%)

*k values are percentage is based on the main question.

*^a^The percentage is based on the 99 total exercise arms.*

*^b^The percentage is based on the 61 total control arms. VO_2_peak, peak oxygen uptake; VO_2_max, maximum oxygen consumption. Items shaded in gray are the core items.*

### Evaluations of Studies Included

For the determination of the overall risk of bias using the Revised Cochrane Risk-of-Bias Tool for Randomized Trial (Higgins et al., 2011), eight studies (12.9%) were of low risk, 39 studies (62.9%) had some concerns, and 17 studies (24.2%) were of high risk. In the *Domain of Bias Arising from the Randomization Process*, 17.2% (*k* = 11) of the studies were of low risk, 75% (*k* = 48) had some concerns, and 7.8% (*k* = 5) were of high risk. In the *Domain of Bias Arising from the Deviations from Intended Interventions*, 64.1% (*k* = 41) of the studies were of low risk, 21.9% (*k* = 14) had some scored some concern, and 14.1% (*k* = 9) were of high risk. In the *Domain of Bias Due to Missing Outcome Data*, 93.8% (*k* = 60) were of low risk, 6.2% of the studies (*k* = 4) had some concerns, and no studies were of high risk. In the *Domain of Bias in Measurement of The Outcome*, 85.5% of the studies (*k* = 55) were of low risk, 14.5% (*k* = 9) had some concerns, and no studies were of high risk. In the *Domain of Bias in Selection of The Reported Result*, 96.8% of the studies (*k* = 62) were of low risk, 3.2% (*k* = 2) of the studies had some concerns, and no studies were high risk. Please see Supplementary Material C for the Risk of Bias scores of qualifying studies.

On the Downs and Black checklist, studies scored averaged 55.6 ± 10 (37.9–79.3%). Of these, 31.3% (*k* = 20) exhibited low methodological quality, most of the studies (68.8%, *k* = 44) exhibited moderate methodological quality (*k* = 44), and no study scored high methodological quality. Meanwhile the average PEH√list study score was 53.9 ± 13.3%. Among the 64 studies, two reached a high checklist study score (81.0 ± 0.02%), 36 reached a moderate checklist study score (62.1 ± 0.08%), and 26 had low study scores (41.2 ± 0.06%). Please see Supplementary Material C for the studies respective PEH√list study score. Based on the Pearson correlation analysis, there was a positive relationship between the Downs and Black checklist score and the PEH√list study score with a Pearson correlation coefficient of 0.325 (*p* = 0.009). Please see Supplementary Material C for the Downs and Black scores of qualifying studies.

## Discussion

The clinical utility of PEH as an antihypertensive lifestyle therapy needs to be better understood partially due to the variations in PEH study designs (MacDonald, 2002; Pescatello et al., 2015a; US Department of Health and Human Services, 2018; de Brito et al., 2019; Fecchio et al., 2020). We developed a 38-item evaluation instrument, the PEH√list, based upon our laboratory (Downs and Black, 1998; Higgins et al., 2011; Ash et al., 2013; Johnson et al., 2014; Hacke et al., 2018; de Brito et al., 2019) and others’ (Hacke et al., 2018; de Brito et al., 2019) experience of performing PEH studies adhering to the contemporary methodological study quality standards of the Cochrane risk of bias tool (Higgins et al., 2011) and Downs and Black checklist for methodological quality (Downs and Black, 1998). We then performed a high-quality systematic review adhering to contemporary standards (Moher et al., 2009) to evaluate qualifying PEH studies with the PEH√list that examined the BP response to acute aerobic exercise.

The average PEH√list study score was 53.94 ± 13.3%. Two studies reached a high checklist study score (81.0 ± 0.02%), 36 reached a moderate checklist study score (62.1 ± 0.08%), and 26 reached a low checklist study scored (41.2 ± 0.06%). Of the three sections of PEH√list, Part 3-Intervention Characteristics (Table 3) had the highest reporting rate of 67.5%, followed by Part 1-Sample Characteristics at 63.6% (Table 1), and Part 2-Study Characteristics at 51.1%. Therefore, Part 2-Study Characteristics (e.g., following professional protocols for the measurement of BP, participant instruction abstaining from caffeine, alcohol, and physical activity) had the most room for improvement.

**FIGURE 2:**
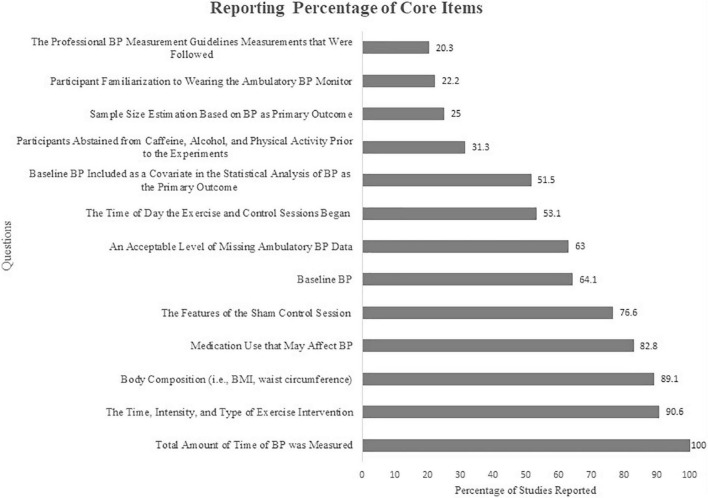
Reporting percentage of PEH√list core items. BP, blood pressure; BMI, body mass index.

The average reporting rates of all PEH√list items were 61.8 ± 31.7%. However, we acknowledge for various reasons it may not be feasible to integrate all the items on the PEH√list into the study protocol. For example, having the same investigator take all BP measurements could be challenging for studies with larger sample sizes. If the accessors are well-trained and follow the same protocol the potential bias for methodological bias would be reduced. Therefore, after careful deliberations, we have identified 13 core items that are fundamental and practical to be controlled for within studies. The PEH√list core items had a reporting rate from 20.3 to 100% with an average rate of 59.2 + 27.9%. These reporting rates are present in Figure 2. The five core items (*n* = 13) that were reported ∼<50% of the time relevant to a given study were: (1) 20% followed standard protocols for measuring BP, such as the American College of Cardiology and American Heart Association guidelines, to ensure the accuracy of BP measurements (Flack and Adekola, 2020); (2) 22% provided an ambulatory BP familiarization session which should be integrated to avoid an alerting reaction to initially wearing the monitor (Thomas et al., 2006; Ash et al., 2013); (3) 25% reported performing a sample size estimation based on the primary BP outcome suggesting many of the qualifying studies may have been underpowered (Guadagnoli and Velicer, 1988); (4) 31% of the studies reported having their participants abstain from caffeine, alcohol, and physical activity that are common PEH confounders (Downs and Black, 1998; Whelton et al., 2018); and (5) 52% of the studies reported they controlled for baseline/pre-exercise BP in their statistical analyses (Eicher et al., 2010). Clearly, the lack of disclosure of these five PEH√list core items and the others shown in Figure 2 indicate a need for improvement in the rigor of PEH studies.

There are some limitations to the current study. First, our review only involved aerobic exercise PEH studies (Johnson et al., 2014; Pescatello et al., 2015a). However, the items within PEH√list are not applicable to only aerobic exercise but to other types of exercise as well. Second, our evaluation of PEH aerobic exercise studies was based on what was reported and may not completely reflect the rigor of the study protocols due to the journal word limitations and the feasibility of implementing certain procedures due to funding limitations, among other reasons. We acknowledge the PEH√list has not been validated; however, the PEH√list and Downs and Black checklist scores had a positive correlation coefficient of 0.325 (*p* = 0.009), indicating the PEH√list can be used as a methodological study quality evaluation tool specifically designed for PEH studies.

Despite these limitations, our study has several strengths. To the best of our knowledge, our study is the first to systematically review the aerobic exercise PEH study methodology. We systematically searched six different databases following PRISMA guidelines. The development of the PEH√list is based on our (Pescatello et al., 1991, 2004, 2007, 2016; Keese et al., 2011; Ash et al., 2013; Headley et al., 2017; Cordeiro et al., 2018; Cilhoroz et al., 2019; Zaleski et al., 2019; Babcock et al., 2020; Farinatti et al., 2021) and others (Hacke et al., 2018; de Brito et al., 2019) long history of performing well-controlled PEH studies (Guadagnoli and Velicer, 1988; Pescatello et al., 1991, 2003, 2004, 2015a, 2017, 2019; Thomas et al., 2006; Moher et al., 2009; Casonatto et al., 2011; Johnson et al., 2014; Matzer et al., 2017) as well as the methodological study quality standards of the Cochrane risk of bias tool (Higgins et al., 2011) and Downs and Black checklist for methodological quality (Downs and Black, 1998). The PEH√list is comprehensive addressing essential study design considerations. Accordingly, investigators even with no prior experience can use our checklist as a template to design their PEH studies.

In conclusion, founded upon a high-quality, contemporary systematic review, we have stringently evaluated aerobic exercise PEH studies with the PEH√list and identified fundamental study design considerations that need improvement. Future researchers should consider using our PEH√list, or at minimum the core items, in conjunction with methodological study quality standards when designing and implementing PEH studies as well as reporting their results.

## Data Availability Statement

The original contributions presented in the study are included in the article/Supplementary Material, further inquiries can be directed to the corresponding author.

## Author Contributions

CD participated in the design of the study, data extraction, and interpretation of details, performed the statistical analysis, and drafted and revised the manuscript critically for important intellectual content. YW participated in the design of the study, data extraction, and interpretation of details and revised the manuscript critically for important intellectual content. LP participated in the design of the study and revised the manuscript critically for important intellectual content and final approval of the version to be published. All authors contributed to the article and approved the submitted version.

## Conflict of Interest

The authors declare that the research was conducted in the absence of any commercial or financial relationships that could be construed as a potential conflict of interest.

## Publisher’s Note

All claims expressed in this article are solely those of the authors and do not necessarily represent those of their affiliated organizations, or those of the publisher, the editors and the reviewers. Any product that may be evaluated in this article, or claim that may be made by its manufacturer, is not guaranteed or endorsed by the publisher.
