# Supplementary material for: Evaluating the Methodological Quality of Postexercise Hypotension Aerobic Exercise Interventions
Source: Front Physiol. 2022 Mar 10;13:851950. doi: 10.3389/fphys.2022.851950 (PMC8960741; doi:10.3389/fphys.2022.851950)
Supplement: Supplementary file 1 [file Table_1.DOCX]

**Supplemental Material A**

| **Evaluation Tool for Studies Examining Postexercise Hypotension (PEH√list)** | |
| --- | --- |
| **Items: Did the Authors report?** | Yes / No / NA |
| **PART I: SAMPLE CHARACTERICS** | |
| 1. Age |  |
| 2. Ethnicity / Race |  |
| 3. Gender / Sex |  |
| 4. BP Classification Scheme Used |  |
| 4a. Followed Professional Guidelines |  |
| *Values at Baseline* |  |
| 5. BP |  |
| 6. Physical Activity Level |  |
| 7. Cardiorespiratory Fitness Level |  |
| 8. Body Mass Index |  |
| 9. Waist Circumference |  |
| 10. Medication Use |  |
| 10a.Type and/or dosage of medication |  |
| 10b. Length of the washout or run-in period |  |
| **PART II: STUDY CHARACTERISTICS** | |
| 11. Performed sample size estimation analysis based on BP as the primary outcome | |
| 12. The allocation sequence |  |
| 12a. The procedure used |  |
| 13. The investigator who performed the BP measurements |  |
| 14. The same investigator performed all BP measurements |  |
| 15. The model of the BP device |  |
| 15a. The same BP device was used through the study for a participant |  |
| 16. Participant abstained from Caffeine prior to intervention |  |
| 16a. Hours participant abstained from Caffeine |  |
| 17. Participant abstained from Alcohol prior to intervention |  |
| 17a. Hours participant abstained from Alcohol |  |
| 18. Participant abstained from Physical Activity prior to intervention |  |
| 18a. Hours participant abstained from Physical Activity |  |
| *Was The BP Response to Exercise Controlled for By Baseline/Pre-Exercise BP* |  |
| **19a. Reported Average= (Average BP post-exercise) minus (Average BP post-control)** |  |
| **19b. Reported Change from baseline= (Average BP post- minus pre-exercise) minus (Average BP post- minus pre-control)** |  |
| *Resting BP Measurement Protocol* |  |
| 20. Location/environment |  |
| 21. Following professional guidelines during BP measurements |  |
| 22. Participant’s position |  |
| 23. Time Lapse from the end of exercise and start of the BP measurements |  |
| 24. Total time of the BP monitoring |  |
| *Ambulatory BP Measurement Protocol* |  |
| 25. Following professional guidelines during BP measurements |  |
| 26. Performing a calibration check |  |
| 27. Including participant familiarization to wearing the ambulatory BP monitor |  |
| 28. Participants were given instruction while wearing the BP monitor |  |
| 29. Time-lapse from the end of exercise and start of BP measurements |  |
| 30. Location/environment |  |
| 31. Disclosing when ABP monitor was attached during the day |  |
| 32. Total time of the BP monitoring |  |
| 33. A specified acceptable level of missing data for ambulatory BP analysis |  |
| **Part III: Intervention Characteristics** |  |
| 34. The time of day the exercise and control sessions began |  |
| 34a. The start of exercise and control sessions were conducted within 3-4 hours of one another |  |
| 35. The location of Exercise |  |
| 36. The temperature that participants exercise in |  |
| 37. The time, intensity, and type of the exercise intervention |  |
| 38. The features of the sham control session |  |
| Index: NA= Not Applicable, BP = Blood pressure, Items shaded in grey are the core items | |

**Supplemental Material B**

References of Included Intervention Studies

1. Ash G, Taylor B, Thompson P, MacDonald H, Lamberti L, Chen M, et al. The antihypertensive effects of aerobic versus isometric handgrip resistance exercise. Journal of hypertension. 2017 Feb;35(2):291-9.

2. Augeri AL, Tsongalis GJ, Van Heest JL, Maresh CM, Thompson PD, Pescatello LS. The endothelial nitric oxide synthase −786 T>C polymorphism and the exercise-induced blood pressure and nitric oxide responses among men with elevated blood pressure. Atherosclerosis. 2008;204(2):e28-34.

3. BHAMMAR DM, ANGADI SS, GAESSER GA. Effects of Fractionized and Continuous Exercise on 24-h Ambulatory Blood Pressure. Medicine and science in sports and exercise. 2012;44(12):2270-6.

4. BHAMMAR D, SAWYER B, TUCKER W, GAESSER G. Breaks in Sitting Time: Effects on Continuously Monitored Glucose and Blood Pressure. Medicine and science in sports and exercise. 2017 Oct;49(10):2119-30.

5. Blanchard BE, Tsongalis GJ, Guidry MA, LaBelle LA, Poulin M, Taylor AL, et al. RAAS polymorphisms alter the acute blood pressure response to aerobic exercise among men with hypertension. Eur J Appl Physiol. 2006;97(1):26-33.

6. Brito LC, Rezende RA, Mendes C, Silva-Junior ND, Tinucci T, Cipolla-Neto J, et al. Separate aftereffects of morning and evening exercise on ambulatory blood pressure in prehypertensive men. J Sports Med Phys Fitness. 2017 -11;58(1-2).

7. Carvalho, Raphael Santos Teodoro de, Pires CMR, Junqueira GC, Freitas D, Marchi-Alves LM. Hypotensive response magnitude and duration in hypertensives: continuous and interval exercise. Arquivos brasileiros de cardiologia. 2015 Mar;104(3):234-41.

8. Casonatto J, Tinucci T, Dourado AC, Polito M. Cardiovascular and autonomic responses after exercise sessions with different intensities and durations. Clinics (São Paulo, Brazil). 2011;66(3):453-8.

9. Chan HH, Burns SF. Oxygen consumption, substrate oxidation, and blood pressure following sprint interval exercise. Applied physiology, nutrition, and metabolism. 2013 Feb;38(2):182-7.

10. Ciolac EG, Guimarães GV, D´Àvila VM, Bortolotto LA, Doria EL, Bocchi EA. Acute effects of continuous and interval aerobic exercise on 24-h ambulatory blood pressure in long-term treated hypertensive patients. International journal of cardiology. 2008;133(3):381-7.

11. Cleroux J, Kouame N, Nadeau A, Coulombe D, Lacourciere Y, Health K. Cléroux1992.

12. Coats AJ, Conway J, Isea JE, Pannarale G, Sleight P, Somers VK. Systemic and forearm vascular resistance changes after upright bicycle exercise in man. The Journal of physiology. 1989 Jun 01,;413(1):289-98.

13. Convertino VA, Adams WC. Enhanced vagal baroreflex response during 24 h after acute exercise. American Journal of Physiology - Regulatory, Integrative and Comparative Physiology. 1991 Mar 01,;260(3):570-5.

14. COOPER AR, GOFF F. Does a single bout of brisk walking reduce ambulatory blood pressure in normotensives or hypertensives? Cardiovascular reviews & reports. 2001;22(5):213-6.

15. Cunha FA, Midgley AW, Soares PP, Farinatti PTV. Postexercise hypotension after maximal short-term incremental exercise depends on exercise modality. Applied physiology, nutrition, and metabolism. 2015;40(6):605-14.

16. Cunha RM, Costa AM, Silva CNF, Póvoa TIR, Pescatello LS, Lehnen AM. Postexercise Hypotension After Aquatic Exercise in Older Women With Hypertension: A Randomized Crossover Clinical Trial. American journal of hypertension. 2018 Jan 12,;31(2):247-52.

17. Cunha R, Macedo C, Araújo S, Santos J, Borges V, Soares Jr A, et al. Subacute Blood Pressure Response in Elderly Hypertensive Women after a Water Exercise Session. High Blood Press Cardiovasc Prev. 2012 Dec;19(4):223-7.

18. Dantas T, Farias Junior L, Frazão D, Silva P, Sousa Junior A, Costa I, et al. A Single Session of Low-Volume High-Intensity Interval Exercise Reduces Ambulatory Blood Pressure in Normotensive Men. Journal of strength and conditioning research. 2017 Aug;31(8):2263-9.

19. de Almeida WS, de Jesus Lima LC, da Cunha RR, Simões HG, Nakamura FY, Campbell CSG. Post-exercise blood pressure responses to cycle and arm-cranking. Science &amp; sports. 2010;25(2):74-80.

20. Enweze L, Oke LM, Thompson T, Obisesan TO, Blakely R, Adams RG, et al. Acute exercise and postexercise blood pressure in African American women. Ethn Dis. 2007;17(4):664.

21. Ferreira AP, Campos BRM, Rodrigues Junior É, Puga GM, Brito CJ. Effects of aerobic and resistance exercise intensities on 24-hours blood pressure in normotensive women. Motriz : Revista de Educação Física. Unesp. 2013 Dec;19(4):681-7.

22. Forjaz CL, Matsudaira Y, Rodrigues FB, Nunes N, Negrão CE. Post-exercise changes in blood pressure, heart rate and rate pressure product at different exercise intensities in normotensive humans. Brazilian journal of medical and biological research. 1998 Oct;31(10):1247-55.

23. Forjaz C, Cardoso CG, Rezk CC, Santaella DF, Tinucci T. Postexercise hypotension and hemodynamics: the role of exercise intensity. J Sports Med Phys Fitness. 2004;44(1):54-62.

24. Forjaz CL, Ortega KC, Santaella DF, Mion Jr D, Negrão CE. Factors affecting post-exercise hypotension in normotensive and hypertensive humans. Blood Press Monit. 2000;5(5):255-62.

25. Fullick S, Morris C, Jones H, Atkinson G. Prior Exercise Lowers Blood Pressure During Simulated Night-Work With Different Meal Schedules. American journal of hypertension. 2009 Aug;22(8):835-41.

26. Hagberg JM, Montain SJ, Martin 3rd WH. Blood pressure and hemodynamic responses after exercise in older hypertensives. J Appl Physiol. 1987;63(1):270-6.

27. Hamer M, Boutcher SH. Impact of moderate overweight and body composition on postexercise hemodynamic responses in healthy men. Journal of human hypertension. 2006 Aug;20(8):612-7.

28. Imazu AA, Goessler KF, Casonatto J, Polito MD. The influence of physical training status on postexercise hypotension in patients with hypertension: a cross-sectional study. Blood pressure monitoring. 2017 Aug;22(4):196-201.

29. Isea JE, Piepoli M, Adamopoulos S, Pannarale G, Sleight P, Coats A. Time course of haemodynamic changes after maximal exercise. Eur J Clin Invest. 1994;24(12):824-9.

30. Jones H, George K, Edwards B, Atkinson G. Exercise Intensity and Blood Pressure During Sleep. International journal of sports medicine. 2009 Feb;30(2):94-9.

31. Jones H, George K, Edwards B, Atkinson G. Is the magnitude of acute post-exercise hypotension mediated by exercise intensity or total work done? Eur J Appl Physiol. 2007 Dec;102(1):33-40.

32. Keese F, Farinatti P, Pescatello L, Monteiro W. A Comparison of the Immediate Effects of Resistance, Aerobic, and Concurrent Exercise on Postexercise Hypotension. Journal of strength and conditioning research. 2011 May;25(5):1429-36.

33. Kingwell BA, Berry KL, Cameron JD, Jennings GL, Dart AM. Arterial compliance increases after moderate-intensity cycling. American journal of physiology. Heart and circulatory physiology. 1997 Nov 01,;273(5):H2186-91.

34. Lehmkuhl LAA, Park S, Zakutansky D, Jastremski CA, Wallace JP. Reproducibility of postexercise ambulatory blood pressure in Stage I hypertension. Journal of human hypertension. 2005 Aug;19(8):589-95.

35. Lund Rasmussen C, Nielsen LL, Linander Henriksen M, Søgaard K, Krustrup P, Holtermann A, et al. Acute effect on ambulatory blood pressure from aerobic exercise: a randomised cross-over study among female cleaners. Eur J Appl Physiol. 2018;118(2):331.

36. Mach C, Foster C, Brice G, Mikat RP, Porcari JP. Effect of Exercise Duration on Postexercise Hypotension. Journal of cardiopulmonary rehabilitation. 2005 Nov;25(6):366-9.

37. Matzer F, Nagele E, Lerch N, Vajda C, Fazekas CC. Combining walking and relaxation for stress reduction-A randomized cross-over trial in healthy adults. Stress and Health. 2017 -08-25;34(2):266.

38. Maya ÁTD, Assunção MJ, Brito CJ, Vieira E, Rosa TS, Pereira FB, et al. High-intensity interval aerobic exercise induced a longer hypotensive effect when compared to continuous moderate. Sport Sci Health. 2018 -03-14;14(2):379.

39. McClean CM, Clegg M, Shafat A, Murphy MH, Trinick T, Duly E, et al. The Impact of Acute Moderate Intensity Exercise on Arterial Regional Stiffness, Lipid Peroxidation, and Antioxidant Status in Healthy Males. Research in sports medicine. 2010 Dec 30,;19(1):1-13.

40. MIYASHITA M, BUMS SF, STENSEL DJ. Accumulating short bouts of brisk walking reduces postprandial plasma triacylglycerol concentrations and resting blood pressure in healthy young men. The American journal of clinical nutrition. 2008;88(5):1225-31.

41. Miyashita M, Burns SF, Stensel DJ. Accumulating short bouts of running reduces resting blood pressure in young normotensive/pre-hypertensive men. Journal of sports sciences. 2011 Nov 01,;29(14):1473-82.

42. Morales-Palomo F, Ramirez-Jimenez M, Ortega JF, Pallares JG, Mora-Rodriguez R. Acute hypotension after high-intensity interval exercise in metabolic syndrome patients. Int J Sports Med. 2017;38(07):560-7.

43. Mota M, Pardono E, Lima L, Arsa G, Bottaro M, Campbell C, et al. Effects of Treadmill Running and Resistance Exercises on Lowering Blood Pressure During the Daily Work of Hypertensive Subjects. Journal of strength and conditioning research. 2009 Nov;23(8):2331-8.

44. Niedermeier M, Grafetstätter C, Hartl A, Kopp M. A Randomized Crossover Trial on Acute Stress-Related Physiological Responses to Mountain Hiking. International journal of environmental research and public health. 2017 Aug 11,;14(8):905.

45. Pardono E, Fernandes MdO, Azevêdo LM, Almeida JAd, Mota MR, Simões HG. Post-exercise hypotension of normotensive young men through track running sessions. Revista brasileira de medicina do esporte. 2015 Jun;21(3):192-5.

46. PARK S, JASTREMSKI CA, WALLACE JP. Time of day for exercise on blood pressure reduction in dipping and nondipping hypertension. Journal of human hypertension. 2005 Aug;19(8):597-605.

47. Park S, Rink L, Wallace J. Accumulation of physical activity leads to a greater blood pressure reduction than a single continuous session, in prehypertension. Journal of hypertension. 2006 Sep;24(9):1761-70.

48. Pescatello LS, Bairos L, VanHeest JL, Maresh CM, Rodriguez NR, Moyna NM, et al. Postexercise hypotension differs between white and black women. The American heart journal. 2003 Feb;145(2):364-70.

49. Pescatello L, Guidry M, Blanchard B, Kerr A, Taylor A, Johnson A, et al. Exercise intensity alters postexercise hypotension. Journal of hypertension. 2004 Oct;22(10):1881-8.

50. Piepoli M, Coats AJ, Adamopoulos S, Bernardi L, Feng YH, Conway J, et al. Persistent peripheral vasodilation and sympathetic activity in hypotension after maximal exercise. Journal of Applied Physiology. 1993 Oct 01,;75(4):1807-14.

51. Piepoli M, Isea JE, Pannarale G, Adamopoulos S, Sleight P, Coats AJ. Load dependence of changes in forearm and peripheral vascular resistance after acute leg exercise in man. The Journal of physiology. 1994 Jul 15,;478(Pt 2):357-62.

52. Pierce DR, Doma K, Raiff H, Golledge J, Leicht AS. Influence of Exercise Mode on Post-exercise Arterial Stiffness and Pressure Wave Measures in Healthy Adult Males. Front Physiol. 2018 -10-01;9.

53. QUINN TJ. Twenty-four hour, ambulatory blood pressure responses following acute exercise: impact of exercise intensity. Journal of human hypertension. 2000;14(9):547-53.

54. Raglin JS, Morgan WP. Influence of exercise and quiet rest on state anxiety and blood pressure. Medicine & Science in Sports & Exercise. 1987.

55. Rueckert PA, Slane PR, Lillis DL, Hanson P. Hemodynamic patterns and duration of post-dynamic exercise hypotension in hypertensive humans. Med Sci Sports Exerc. 1996;28(1):24-32.

56. Santaella D, Araújo E, Ortega K, Tinucci T, Mion D, Negrão C, et al. Aftereffects of Exercise and Relaxation on Blood Pressure. Clinical journal of sport medicine. 2006 Jul;16(4):341-7.

57. Santana HAP, Moreira SR, Neto WB, Silva CB, Sales MM, Oliveira VN, et al. The higher exercise intensity and the presence of allele I of ACE gene elicit a higher post-exercise blood pressure reduction and nitric oxide release in elderly women: an experimental study. BMC cardiovascular disorders. 2011 Dec 02,;11(1):71.

58. Schuster-Decker R, Foster C, Porcarp JP, -&#39; &#39;, Margaret A Mahe~. cute Effects of Dynarnic Exercise and Nutritional Supplementation on Blood Pressure in Mildly Hypertensive Patients.

59. Silva TFd, França, Ana Carla Lima de, Souza MFd, Silva AS. A single session of active video game play promotes post-exercise hypotension in hypertensive middle-aged subjects. Human movement. 2018;2018(2):82-9.

60. Someya N, Ikemura T, Hayashi N. Effect of preceding exercise on cerebral and splanchnic vascular responses to mental task. Journal of physiological anthropology. 2012 Jun 25,;31(1):17.

61. Taylor-Tolbert NS, Dengel DR, Brown MD, Mccole SD, Pratley RE, Ferrell RE, et al. Ambulatory blood pressure after acute exercise in older men with essential hypertension. American Journal of Hypertension. 2000;13(1):44.

62. WALLACE JP, BOGLE PG, KING BA, KRASNOFF JB, JASTREMSKI CA. The magnitude and duration of ambulatory blood pressure reduction following acute exercise. Journal of human hypertension. 1999;13(6):361-6.

63. Wallace JP, Bogle PG, King BA, Krasnoff JB, Jastremski CA. A Comparison of 24-h Average Blood Pressures and Blood Pressure Load Following Exercise. American Journal of Hypertension. 1997 -07;10(7):728.

64. Williamson JW, McColl R, Mathews D. Changes in regional cerebral blood flow distribution during postexercise hypotension in humans. J Appl Physiol. 2004;96(2):719-24.

**Supplemental Material C**

| Cochrane Risk of Bias | | | | | | | |  | Downs & Black | PEH√list |
| --- | --- | --- | --- | --- | --- | --- | --- | --- | --- | --- |
| Author | Year Published | Domain #1 | Domain #2 | Domain #3 | Domain #4 | Domain #5 | Overall Score |  | Score | Study Score |
| Almedia et. al. | 2010 | SC | LR | LR | LR | LR | SC |  | 55.2% | 72.4% |
| Ash et. al. | 2017 | LR | SC | LR | LR | LR | SC |  | 79.3% | 57.6% |
| Augeri et. al. | 2008 | LR | LR | LR | LR | LR | LR |  | 65.5% | 60.6% |
| Bhammar et. al. | 2017 | SC | SC | LR | LR | LR | SC |  | 72.4% | 51.5% |
| Bhammer et. al. | 2012 | SC | LR | LR | LR | SC | SC |  | 65.5% | 45.5% |
| Blanchard et. al. | 2006 | LR | LR | LR | LR | LR | LR |  | 48.3% | 51.5% |
| Brito et. al. | 2017 | SC | SC | LR | LR | LR | SC |  | 55.2% | 51.5% |
| Carvalho et. al. | 2015 | SC | SC | LR | LR | LR | SC |  | 41.4% | 24.2% |
| Casonatto et. al. | 2011 | LR | LR | LR | LR | LR | LR |  | 69.0% | 75.9% |
| Chan et. al. | 2013 | SC | SC | LR | SC | LR | SC |  | 58.6% | 65.5% |
| Ciolac et. al. | 2008 | SC | HR | SC | LR | LR | HR |  | 55.2% | 42.4% |
| Cleroux et. al. | 1992 | SC | LR | LR | LR | LR | SC |  | 55.2% | 54.5% |
| Coats et. al. | 1989 | SC | SC | LR | LR | LR | SC |  | 44.8% | 48.5% |
| Convertino et. al. | 1991 | SC | HR | LR | LR | LR | HR |  | 48.3% | 42.4% |
| Cooper et. al. | 2001 | SC | LR | LR | LR | LR | SC |  | 69.0% | 33.3% |
| Cunha et. al. | 2012 | SC | SC | LR | LR | LR | SC |  | 58.6% | 62.1% |
| Cunha et. al. | 2015 | SC | LR | LR | LR | LR | SC |  | 48.3% | 65.5% |
| Cunha et. al. | 2018 | LR | SC | SC | LR | LR | SC |  | 58.6% | 45.5% |
| Dantas et. al. | 2017 | SC | LR | LR | LR | LR | SC |  | 72.4% | 45.5% |
| Da Silva et. al. | 2018 | SC | LR | LR | LR | LR | SC |  | 75.9% | 72.4% |
| Enweze et. al. | 2007 | SC | HR | LR | LR | LR | HR |  | 48.3% | 65.5% |
| Ferreira et. al. | 2013 | SC | LR | LR | LR | LR | SC |  | 51.7% | 69.0% |
| Forjaz et. al. | 1998 | HR | LR | LR | SC | LR | HR |  | 48.3% | 65.5% |
| Forjaz et. al. | 2000 | HR | LR | LR | LR | LR | HR |  | 48.3% | 42.4% |
| Forjaz et. al. | 2004 | SC | SC | LR | LR | LR | SC |  | 51.7% | 52.6% |
| Fullick et. al. | 2009 | SC | LR | LR | LR | LR | SC |  | 69.0% | 82.8% |
| Hagberg et. al. | 1987 | SC | LR | LR | SC | LR | SC |  | 41.4% | 51.7% |
| Hamer et. al. | 2006 | SC | LR | LR | LR | HR | HR |  | 41.4% | 58.6% |
| Isea et. al. | 1994 | LR | LR | LR | LR | LR | LR |  | 44.8% | 41.4% |
| Imazu et. al. | 2017 | SC | LR | LR | LR | LR | SC |  | 62.0% | 54.5% |
| Jones et. al. | 2007 | SC | LR | LR | LR | LR | SC |  | 69.0% | 79.3% |
| Jones et. al. | 2009 | HR | LR | LR | LR | LR | HR |  | 58.6% | 57.9% |
| Keese et. al. | 2011 | SC | LR | LR | LR | LR | SC |  | 51.7% | 54.5% |
| Kingwell et. al. | 1997 | SC | LR | SC | LR | LR | SC |  | 58.6% | 69.0% |
| Lehmkuhl et. al. | 2005 | HR | SC | LR | LR | LR | HR |  | 41.4% | 30.3% |
| Mach et. al. | 2005 | SC | SC | LR | LR | HR | HR |  | 55.2% | 62.1% |
| Matzer et. al. | 2017 | LR | HR | LR | LR | LR | HR |  | 75.9% | 62.1% |
| Maya et. al. | 2018 | SC | LR | LR | SC | LR | HR |  | 62.1% | 75.9% |
| McClean et. al. | 2010 | SC | LR | LR | LR | LR | SC |  | 51.7% | 58.6% |
| Miyashita et. al. | 2008 | SC | LR | LR | SC | LR | SC |  | 58.6% | 72.4% |
| Miyashita et. al. | 2011 | SC | LR | LR | SC | LR | SC |  | 58.6% | 72.4% |
| Morales-Palomo et. al. | 2017 | SC | HR | LR | LR | LR | HR |  | 55.2% | 44.8% |
| Mota et. al. | 2009 | SC | LR | LR | SC | LR | SC |  | 51.7% | 72.4% |
| Niedermeier et. al. | 2017 | SC | HR | LR | LR | LR | HR |  | 69.0% | 48.3% |
| Pardono et. al. | 2015 | SC | LR | LR | SC | SC | SC |  | 37.9% | 44.8% |
| Park et. al. | 2005 | SC | LR | LR | LR | LR | SC |  | 55.2% | 42.4% |
| Park et. al. | 2006 | SC | LR | LR | LR | LR | SC |  | 65.5% | 39.4% |
| Pescatello et. al. | 2003 | SC | LR | SC | LR | LR | SC |  | 51.7% | 54.5% |
| Pescatello et. al. | 2004 | LR | LR | LR | LR | LR | LR |  | 72.4% | 55.3% |
| Piepoli et. al. | 1993 | LR | LR | LR | LR | LR | LR |  | 48.3% | 44.8% |
| Piepoli et. al. | 1993 | LR | LR | LR | LR | LR | LR |  | 48.3% | 51.7% |
| Pierce et. al. | 2018 | LR | LR | LR | LR | LR | LR |  | 62.1% | 65.5% |
| Quinn et. al. | 2000 | SC | HR | LR | LR | LR | HR |  | 48.3% | 45.5% |
| Raglin et. al. | 1987 | SC | HR | LR | LR | LR | HR |  | 37.9% | 37.9% |
| Rasmussen et. al. | 2018 | SC | LR | LR | LR | LR | SC |  | 44.8% | 36.4% |
| Rueckert et. al. | 1996 | SC | SC | LR | LR | LR | SC |  | 62.1% | 47.4% |
| Santaella et. al. | 2006 | SC | LR | LR | LR | LR | SC |  | 51.7% | 72.4% |
| Santana et. al. | 2011 | SC | HR | LR | LR | LR | HR |  | 41.4% | 51.7% |
| Schuster-Decker et. al. | 2002 | SC | LR | LR | LR | LR | SC |  | 51.7% | 41.4% |
| Someya et. al. | 2012 | HR | SC | LR | LR | LR | HR |  | 51.7% | 51.7% |
| Taylor-Tolbert et. al. | 2000 | SC | LR | LR | LR | LR | SC |  | 51.7% | 42.4% |
| Wallace et. al. | 1997 | SC | LR | LR | LR | LR | SC |  | 48.3% | 33.3% |
| Wallace et. al. | 1999 | SC | LR | LR | SC | LR | SC |  | 55.2% | 33.3% |
| Williamson et. al. | 2003 | SC | SC | LR | LR | LR | SC |  | 51.7% | 48.3% |
